# Supplementary material for: TMS-Induced Central Motor Conduction Time at the Non-Infarcted Hemisphere Is Associated with Spontaneous Motor Recovery of the Paretic Upper Limb after Severe Stroke
Source: Brain Sci. 2021 May 15;11(5):648. doi: 10.3390/brainsci11050648 (PMC8157217; doi:10.3390/brainsci11050648)
Supplement: Supplementary file 1 [file brainsci-11-00648-s001.zip › brainsci-1192017-supplementary.pdf]

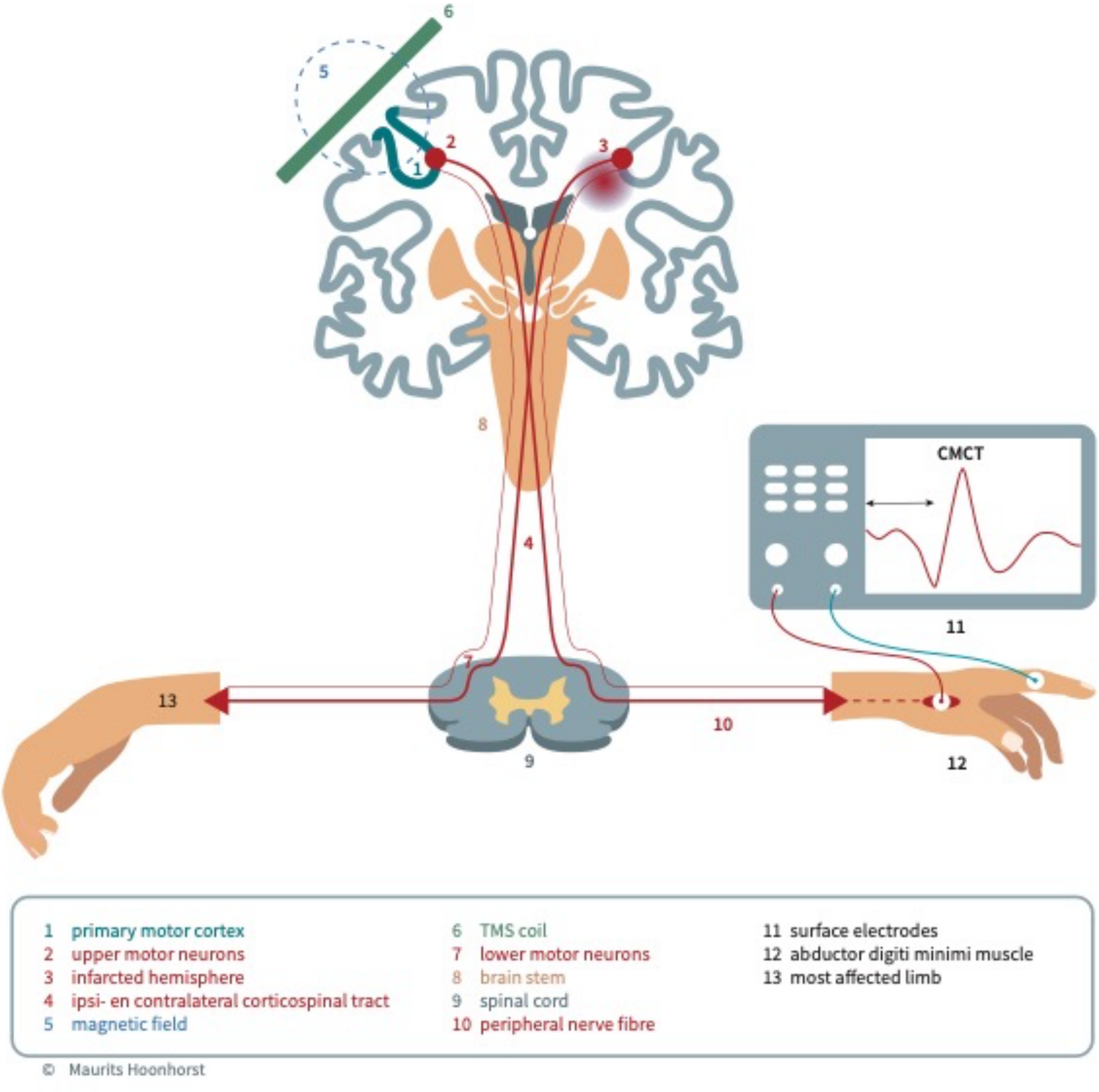

Note. *Simplified mechanism of action of Transcranial Magnetic Stimulation (TMS) of the motor cortex at the non-infarcted hemisphere as performed in our study.*
TMS applied over the motor cortex preferentially excite interneurons of the cortical surface. This placement leads to a transsynaptic activation of pyramidal cells evoking descending volleys in the contralateral corticospinal tract. Motoneuron activation in response to TMS-induced corticospinal volleys leads to a contraction in the target muscle, evoking a motor-evoked potential on electromyography recorded by using surface electrodes applied over the muscle belly of the adductor digit minimi muscle of the less affected limb. Thereafter, CMCT at the non-infarcted hemisphere can be calculated.
